# A CNTNAP1 Missense Variant Associated With Laryngeal Paralysis and Polyneuropathy in Young Great Dane Dogs

**DOI:** 10.1111/jvim.70185

**Published:** 2025-07-07

**Authors:** G. Diane Shelton, Missy C. Carpentier, Y. Michael Kimura, Ling T. Guo, Katie M. Minor

**Affiliations:** ^1^ Department of Pathology, School of Medicine University of California San Diego LaJolla California USA; ^2^ Minnesota Veterinary Neurology Columbus Minnesota USA; ^3^ BluePearl Specialty and Emergency Pet Hospital Tampa Florida USA; ^4^ Department of Veterinary Clinical Sciences, College of Veterinary Medicine University of Minnesota Saint Paul Minnesota USA

**Keywords:** canine, genetic disease, lower motor neuron disease, peripheral nerve, weakness

## Abstract

**Background:**

Major genetic risk loci and causative mutations classified as LPN1 (Leonberger polyneuropathy type 1), LPN2 (Leonberger polyneuropathy type 2), and LPPN3 (Laryngeal paralysis polyneuropathy type 3) have been identified in Leonberger and Saint Bernard dogs with laryngeal paralysis and polyneuropathy (LPPN). Other large breed dogs, including the Great Dane, can present clinically with LPPN, and this breed previously was identified as a carrier of the LPPN3 variant. To date, homozygosity for this variant has not been identified in Great Dane dogs.

**Interventions:**

Results of neurological examination, electrodiagnostic testing, and muscle and nerve biopsy samples were consistent with lower motor neuron disease associated with axonal degeneration and large nerve fiber loss in two young Great Dane dogs with gait abnormalities and respiratory difficulty. TaqMan genotyping for the three known LPPN variants confirmed a homozygous missense variant in *CNTNAP1*, the variant associated with LPPN3.

**Conclusion and Clinical Relevance:**

A homozygous missense *CNTNAP1* variant (p.G937E, XP_548083.3) has been confirmed in young Great Dane dogs that should expand the genetic testing available for LPPN in this breed and aid in the direction of breeding programs. Both dogs were euthanized because of progression of clinical signs approximately 6 months after the original diagnosis.

**Abbreviations**
CSF: cerebrospinal fluid
LPN1: leonberger polyneuropathy type 1
LPN2: leonberger polyneuropathy type 2
LPPN: laryngeal paralysis polyneuropathy
LPPN3: laryngeal paralysis polyneuropathy type 3

## 1 Introduction

The clinical syndrome of laryngeal paralysis and polyneuropathy (LPPN) affects several large and giant breed dogs with both early and late onset of clinical signs [1, 2, 3, 4]. It is difficult to precisely classify these polyneuropathies clinically because they have a similar presentation. Neurological examination confirms lower motor neuron weakness, electrodiagnostic examinations are consistent with axonal loss, and muscle and nerve biopsies confirm a pattern of denervation in muscle biopsy samples with axonal degeneration evident in peripheral nerve biopsy samples. With the advent of advances in molecular testing at a reasonable cost, it is now possible to precisely classify these polyneuropathies based on the genetic variant. In the past several years, a variant in *ARHGEF10* (LPN1, OMIA 001917‐9615) has been confirmed in Leonberger and Saint Bernard dogs with LPPN [5] and a second variant identified in *GJA9* (LPN2, OMIA 001917‐9615) in the Leonberger [6]. Most recently, a third missense homozygous variant in *CNTNAP1* (LPPN3, OMIA 002301–9615) was identified in LPPN‐affected Leonberger, Saint Bernard, and Labrador retriever breeds, with heterozygotes identified in several additional breeds including the Great Dane dog [7]. Since this last report, two Great Dane dogs with early onset polyneuropathy were evaluated and tested for the LPPN3 missense variant. Both dogs were confirmed homozygous for the LPPN3 variant.

## 2 Case 1: History, Diagnostic Testing, and Outcome

A 2‐year‐old male, Great Dane dog was evaluated for a change in bark and knuckling on all four limbs. The owner of the dog was a veterinarian who kept a detailed log on the clinical progression. The owner first noticed weakness of the left thoracic limb at approximately 1.5 years of age with occasional knuckling on the limb. Over the next several months, stamina on walks and hikes decreased, with a change in the breathing pattern, including inspiratory stridor. A laryngeal examination was performed under sedation before anesthesia for neutering confirmed laryngeal paralysis. Weakness progressed over the next few months with more consistent paresis in the left thoracic limb and both pelvic limbs, as well as the development of generalized muscle atrophy. A CBC and serum biochemistry profile were performed and results were unremarkable. Total l‐thyroxine (T4) concentration, free T4 concentration by equilibrium dialysis, and thyroid‐stimulating hormone concentration were normal, and an acetylcholine receptor antibody titer was within the reference range.

The dog was referred to Minnesota Veterinary Neurology, Columbus, MN for examination by a board‐certified veterinary neurologist. A complete physical examination was performed, with inspiratory stridor noted on excitement and generalized muscle atrophy. On neurologic evaluation, the dog was ambulatory with tetraparesis and prominent distal weakness in all four limbs that was most severe in the pelvic limbs. Ataxia was not noted. The cranial nerve examination was normal and spinal hyperesthesia not noted. Because the dog was anxious and difficult to handle, further neurological evaluation could not be performed.

Clinical signs were indicative of lower motor neuron disease, with a degenerative breed‐associated polyneuropathy as the primary differential diagnosis. Three‐view thoracic radiographs were performed before general anesthesia and were normal. Results of complete neuraxis magnetic resonance imaging (MRI) and cerebrospinal fluid evaluation were normal. Electrodiagnostic testing was not available. Unfixed chilled and formalin‐fixed biopsy samples collected from the cranial tibial muscle and a formalin‐fixed biopsy sample from the common peroneal nerve were submitted to the Comparative Neuromuscular Laboratory, University of California San Diego, La Jolla, CA, by an overnight service under refrigeration. Recovery from general anesthesia was uneventful and the dog was discharged the same day.

**FIGURE 1:**
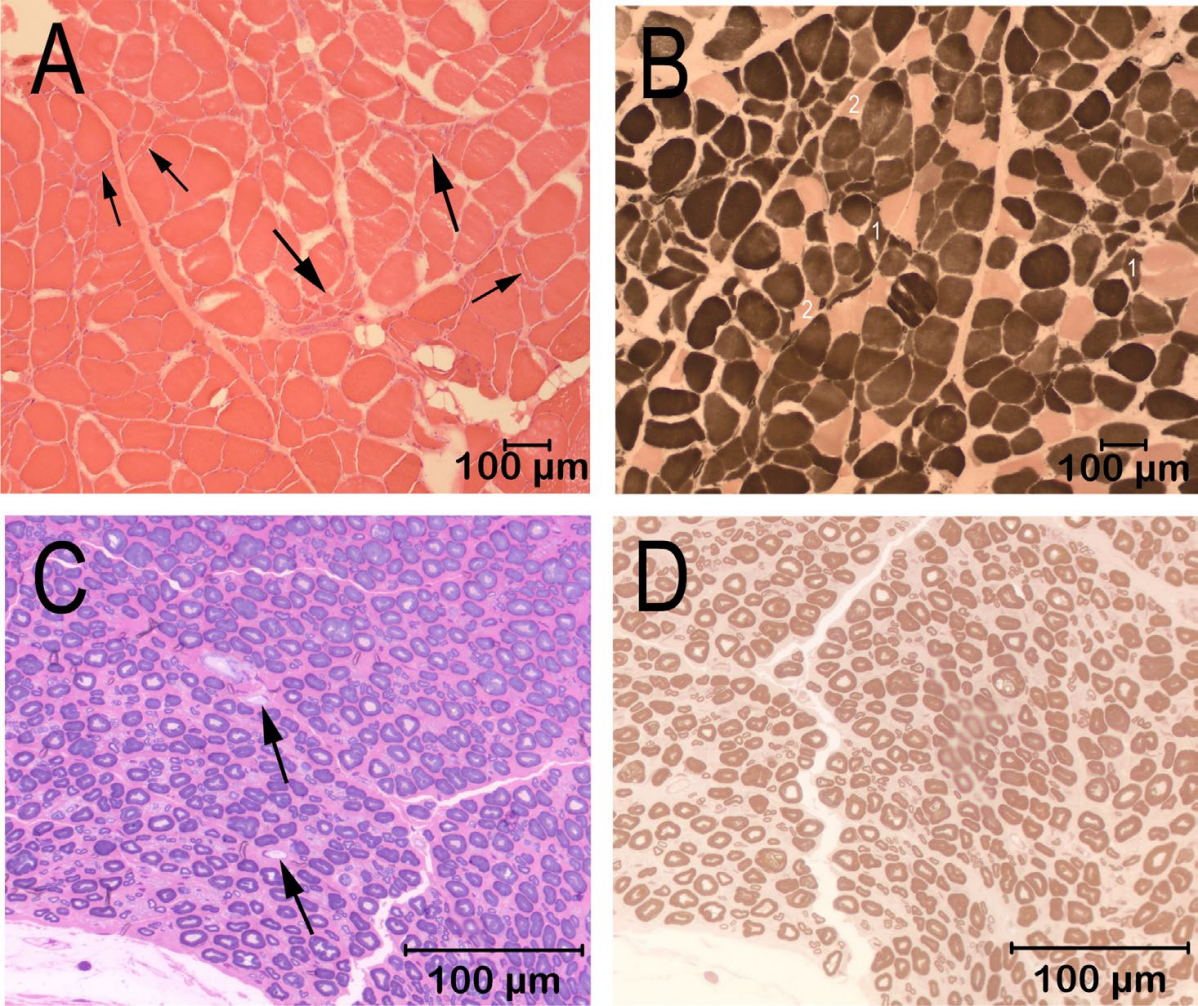
Cryosections from the cranial tibial muscle are shown in A (H&E stain) and B (myofibrillar ATPase reaction for fiber typing at pH 4.3) where type 1 fibers are darkly stained, and type 2 fibers are lightly stained. Variability in myofiber size was present with scattered and small groups of atrophic fibers having an angular to anguloid shape (A, large arrows point to small groups of atrophic fibers having an angular to anguloid shape and small arrows point to individual atrophic fibers). The atrophic fibers were of both types (B). A marked type 1 fiber predominance was present. A fixed biopsy from the peroneal nerve was evaluated in 1 μm resin sections (C, D). Sections were stained for light microscopy with toluidine blue for general morphologic features (C) or with the myelin stain paraphenylenediamine (D). Nerve fiber density was reduced with mild nerve fiber loss and an increased population of small caliber fibers. Sporadic myelin ovoids are highlighted by arrows and shown in (C). The pathological changes are consistent with active polyneuropathy associated with nerve fiber loss resulting from axonal degeneration.

Cryosections from the cranial tibial muscle were evaluated by a standard panel of histochemical stains and reactions, including the myofibrillar adenosine triphosphatase (ATPase) reactions for muscle fiber typing (Figure 1). A marked variability in myofiber size was present, with fiber diameters ranging from 9 to 144 μm (Figure 1A). Scattered and small groups of atrophic fibers were present, with atrophic fibers having an angular to anguloid shape and of both fiber types (Figure 1A,B). A marked type 1 fiber predominance was present, without obvious fiber type grouping (Figure 1B). Intramuscular nerve branches were not present in the sections for evaluation. A fixed biopsy sample from the peroneal nerve was evaluated in 1‐μm resin sections and stained with toluidine blue for general morphologic features (Figure 1C) and with the myelin stain paraphenylenediamine (Figure 1D). Nerve fiber density was subjectively decreased, with large fiber loss and a larger than expected population of small caliber fibers (Figure 1C). Sporadic myelin ovoids were identified, consistent with a late stage of axonal degeneration (Figure 1C, arrows). Myelin sheath thickness was appropriate for the axon diameter (Figure 1D). The pathological changes supported a diagnosis of active polyneuropathy associated with nerve fiber loss resulting from axonal degeneration.

Genotyping using custom TaqMan assays (ANXG7XR, ANRWXTN, and ANXG4XP) for the known LPN1, LPN2, and LPPN3 variants (Applied Biosystems, Thermo Fisher Scientific Inc., Waltham, MA, USA), respectively, was performed at the University of Minnesota, St. Paul, MN. The dog was negative for the LPN1 and LPN2 variants but was homozygous positive for the missense variant *CNTNAP1* p.G937E. A male symptomatic littermate clinically diagnosed with laryngeal paralysis and tetraparesis but not evaluated in our laboratory subsequently was tested for the LPPN3 variant and was similarly homozygous positive. Three other asymptomatic related dogs were tested and shown to be negative or carriers. All three dogs were either littermates of the affected dog or a half‐sibling to the sire and thus were old enough that clinical signs would likely have been apparent if the dogs were affected.

Clinical signs progressed over the next several months with the additional diagnosis of atypical hypoadrenocorticism after gastrointestinal signs developed and further diagnostic testing confirmed atypical hypoadrenocorticism. Treatment was initiated with physiological doses of prednisone. Muscle atrophy progressed, affecting the masticatory muscles and all limb muscles. Exercise tolerance was poor, but the dog was still able to go on short, slow walks with frequent stops. As mobility decreased, the laryngeal paralysis worsened. Because of a poor quality of life, the dog was euthanized approximately 6 months after the original diagnosis.

## 3 Case 2: History, Diagnostic Testing, and Outcome

A 2‐year‐old male neutered Great Dane dog was presented to a board‐certified veterinary neurologist at BluePearl Specialty and Emergency Pet Hospital, Tampa, FL, for evaluation of progressive weakness and lower motor neuron signs, mild postural reaction deficits in the thoracic limbs, and mild generalized ataxia beginning at 22 months of age. The neurological examination confirmed localization to the lower motor neuron with decreased spinal reflexes. Cranial nerve examination was normal. Inspiratory stridor was not noted. An airway evaluation was not performed, but no abnormalities were reported upon intubation for anesthesia for further evaluation. Given the young age, an MRI (thoracolumbar to lumbosacral) evaluation was performed because a developmental myelopathy was being considered. Possible meningeal thickening in the lumbar region was noted. Cerebrospinal fluid was normal. After the MRI, an electromyographic examination was performed and showed diffuse fibrillation potentials, positive sharp waves, and a few runs of complex repetitive discharges throughout the pelvic intumescence distribution. These changes were most prominent in the distal limb muscles. Paraspinal muscles and thoracic limb muscles did not show clinically relevant changes.

**FIGURE 2:**
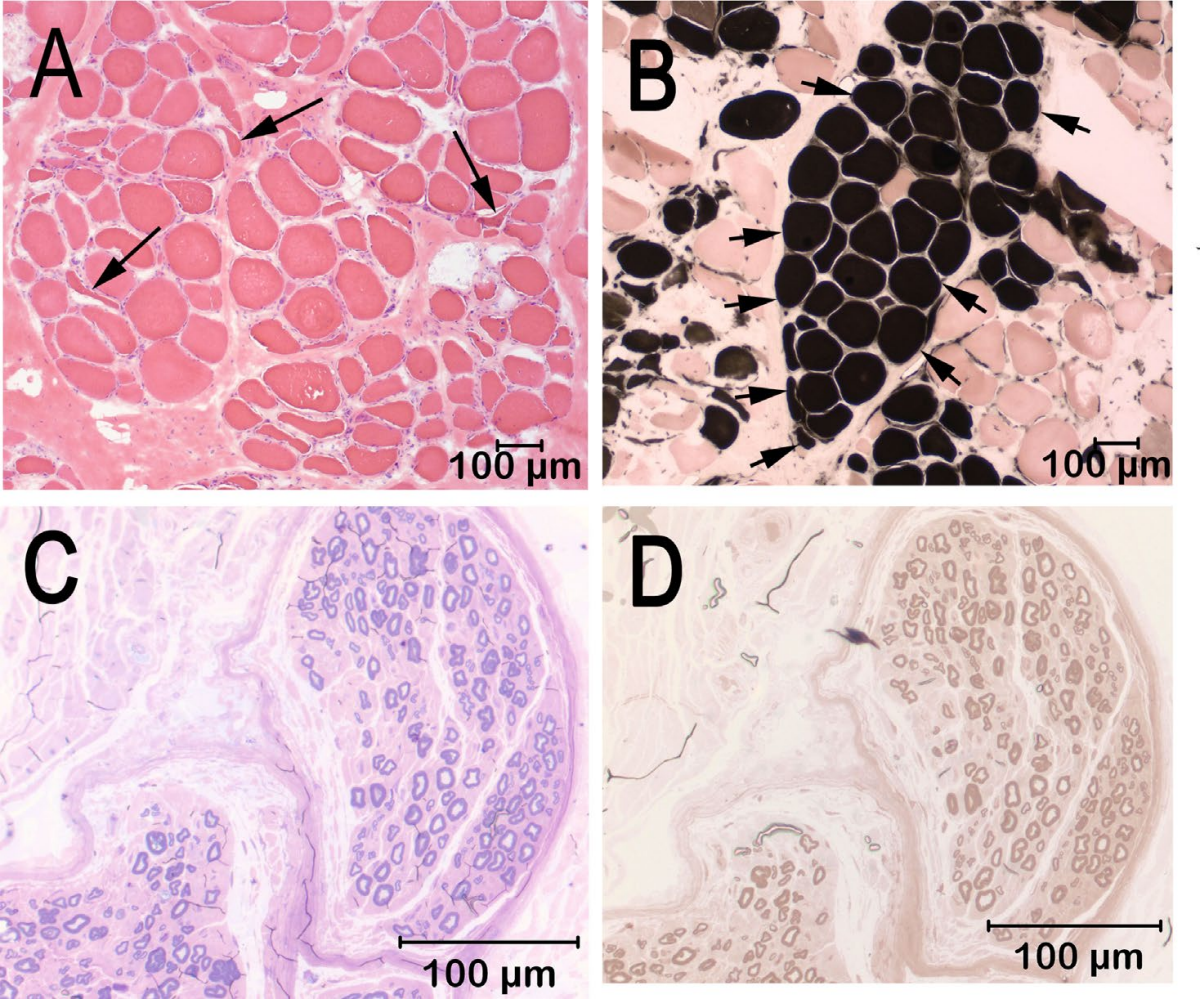
Cryosections from the cranial tibial muscle are shown in A (H&E stain) and in B (myofibrillar ATPase reaction at pH 4.3) with type 1 fibers darkly stained and type 2 fibers lightly stained. A marked variability in myofiber size was present with scattered atrophic fibers having an anguloid to angular shape (A, arrows highlight atrophic fibers) and of both fiber types (B). Different from Case 1, prominent type 1 fiber grouping consistent with chronicity and reinnervation is illustrated (B, multiple arrows outline the large group of type 1 fibers). A fixed biopsy from the peroneal nerve was evaluated in 1 μm resin sections (C, D). Sections were stained for light microscopy using toluidine blue for general morphologic features (C) or with the myelin stain paraphenelenediamine (D). Nerve fiber density is markedly reduced with large nerve fiber loss and endoneurial fibrosis. Sporadic nerve fibers showed early axonal degeneration (C, D). Clusters of small thinly myelinated fibers were observed, suggesting attempted axonal regeneration. Chronic polyneuropathy was diagnosed with fiber type grouping in the cranial tibial muscle and axonal degeneration in the peroneal nerve.

After electrodiagnostic testing, unfixed chilled and formalin‐fixed biopsy samples were collected from the cranial tibial muscle and a formalin fixed‐biopsy sample was collected from the peroneal nerve. The biopsy samples were submitted to the Comparative Neuromuscular Laboratory, La Jolla, CA by an overnight service under refrigeration. Marked variability in myofiber size was present in the cranial tibial muscle with fiber diameters ranging from 11 to 152 μm. Scattered and small groups of atrophic fibers had an anguloid to angular shape and were of both fiber types (Figure 2A). Prominent type 1 fiber grouping was identified (Figure 2B). Intramuscular nerve branches were not present for evaluation. No inflammation, necrosis, fibrosis, fiber loss, organisms, or other specific cytoarchitectural abnormalities were observed. A fixed biopsy sample from the peroneal nerve was processed in 1‐μm resin sections and stained with toluidine blue (Figure 2C) and paraphenylenediamine (Figure 2D). One large and one medium‐sized nerve fascicle were present in the sections. Nerve fiber density was mildly decreased in the large fascicle and markedly decreased in the medium‐sized fascicle with sporadic nerve fibers showing early axonal degeneration. Subperineurial edema was present in the medium‐sized fascicles. Small caliber thinly myelinated fibers were observed, suggesting nerve sprouts and attempted axonal regeneration. Chronic polyneuropathy was diagnosed with fiber type grouping in the cranial tibial muscle and axonal degeneration in the peroneal nerve.

After the above examinations, the dog was placed on prednisone 30 mg PO q24h tapered off over three weeks and gabapentin 200 mg PO q8‐12h as needed for discomfort while awaiting biopsy results. Total T4, free T4, and TSH concentrations were normal. Mild functional improvement was noted, initially with fewer deficits. The dog progressively worsened and was euthanized approximately 6 months after the original diagnosis. Samples of DNA extracted from archived frozen muscle were submitted to the University of Minnesota for TaqMan genotyping as described for Case 1. The dog was homozygous positive for the *CNTNAP1 p.G937E* variant.

## 4 Discussion

In this report we confirm the homozygous missense CNTNAP*1* p.G937E variant in two young Great Dane dogs, both confirmed clinically with lower motor neuron weakness and histologically with muscle and peripheral nerve biopsy samples. Heterozygotes for this variant in the Great Dane breed had previously been reported but homozygotes were not identified at the time of publication [7]. The heterozygotes support the presence of the variant in this breed and the possibility that homozygosity and clinical disease could exist. Our findings suggest that testing for the LPPN3 variant should be performed in any young Great Dane dog with clinical signs of polyneuropathy even if laryngeal paralysis is not noted at the time of diagnosis. Case 1 had laryngeal paralysis at the time of the original diagnosis. Case 2 did not show inspiratory stridor or evidence of laryngeal abnormalities at the time of intubation for diagnostic testing requiring anesthesia. In a validation cohort from the original study [7] that included three large breeds with available health information (Leonberger, Saint Bernard, and Labrador retriever), the variant was not present in a homozygous state in any dog apparently not clinically affected with LPPN. Based on the results of our study, it is likely that laryngeal paralysis could develop over time in Case 2 or could have been detected if more focused studies were performed.

The clinical phenotype in the LPPN3 affected Great Dane dogs is similar to that of LPPN affected Leonberger, Saint Bernard, and Labrador retriever dogs, although some variability in age of onset can be found among breeds. Dogs affected with the *CNTNAP1* variant were often juvenile, but an age of onset up to 6 years of age has been described in the Leonberger dog, and up to 12 years of age in the Labrador retriever [7]. The clinical presentation of inspiratory stridor resulting from laryngeal paralysis, lower motor neuron weakness, and neurogenic muscle atrophy is consistent among breeds and variants. Genotyping for the known LPPN variants is an important tool for accurate classification of polyneuropathy and for breeding programs.

The variants identified to date for LPPN likely do not account for all cases in large and giant breeds and it is probable that additional pathogenic variants will be identified in the future. Genetic characterization of variants supported by clinical evidence of polyneuropathy established by neurological examination, electrodiagnostic testing and muscle and nerve biopsies, should enable accurate classification of these breed‐associated polyneuropathies and lead to more widely available genetic testing.

The *CNTNAP1 p.G937E* variant is present at an allele frequency of 0.008 in the Dog 10 k Consortium Database of whole genome sequences from 1987 canids (331 breeds and 63 wolves) [8] and 0.017 in the Dog Biomedical Variant Database Consortium database [9] of whole genome sequences from 813 canids (137 breeds and 9 wolves). Of note, homozygosity for the *CNTNAP1* variant is most prevalent in the English bulldog, not considered a large or giant breed or a breed typically associated with LPPN. It is not clear if LPPN may be present but underdiagnosed in the breed because of presumptive brachycephalic airway syndrome or if the smaller stature of the breed results in a geriatric onset. American Kennel Club breed standard heights have been described for Saint Bernards, Leonbergers, Great Danes, and for Labrador retrievers with comparison to age of onset of peripheral neuropathy (Table 1, https://www.akc.org/dog‐breeds, accessed March 6, 2025). For comparison to the other breeds, the average height for the English bulldog is smaller for both sexes. It is possible that the *CNTNAP1* variant is nerve fiber length‐dependent for age of onset, and screening English bulldogs with brachycephalic airway syndrome for the *CNTNAP1* variant may be warranted. Similar correlations between height and risk for developing peripheral neuropathy have been described in people [10] and in horses [11, 12].

**TABLE 1:** Relationship of breed height and age of onset of LPPN in various dog breeds.

Breed	Male height (in.)	Female height (in.)	Average age of onset (years)	Age of onset range (years)
Great Dane	30–32	28–30	1.7	1.5–1.8
Saint Bernard	28–30	26–28	1.6	0.25–3
Leonberger	28–31.5	25.5–29.5	3.2	0.75–6
Labrador retriever	22.5–24.5	21.5–23‐5	6.9	2.5–12
English bulldog	14–15	14–15	Unknown	Unknown

*Note:* American Kennel Club breed standards. Height measured in inches at the withers (highest point of the shoulders; https://www.akc.org/dog‐breeds, accessed March 6, 2025).

## 5 Conclusion and Clinical Importance

Here we confirm homozygosity for the *CNTNAP1* variant in Great Dane dogs with polyneuropathy supported by electrodiagnostic and tissue biopsy sample investigations. This finding adds the Great Dane to the list of breeds with homozygosity for this variant and widens the spectrum of genetic testing available for breeds with LPPN. Our findings also suggest homozygosity may be present in other breeds. Genotyping breeds of dogs listed as heterozygous for the *CNTNAP1* variant [7] and showing clinical signs of LPPN is warranted.

## Disclosure

Authors declare no off‐label use of antimicrobials.

## Ethics Statement

The authors declare no institutional animal care and use committee or other approval was needed. Authors declare human ethics approval was not needed.

## Conflicts of Interest

The University of Minnesota offers genotyping tests for polyneuropathy‐associated variants in their laboratory, and proceeds from these tests fund ongoing genetic research in dogs. The other authors declare no conflicts of interest.
